# Bootstrap ARDL on Health Expenditure, CO2 Emissions, and GDP Growth Relationship for 18 OECD Countries

**DOI:** 10.3389/fpubh.2019.00324

**Published:** 2019-11-20

**Authors:** Chien-Ming Wang, Hsin-Pei Hsueh, Fangjhy Li, Cheng-Feng Wu

**Affiliations:** ^1^School of Economics and Trade, Hubei University of Economics, Wuhan, China; ^2^Institute for Development of Cross-Strait Small and Medium Enterprise, Hubei University of Economics, Wuhan, China; ^3^School of Finance, Hubei University of Economics, Wuhan, China; ^4^School of Business Administration, Hubei University of Economics, Wuhan, China; ^5^Research Center of Hubei Logistics Development, Hubei University of Economics, Wuhan, China; ^6^Development and Financial Security Research Center, Hubei University of Economics, Wuhan, China

**Keywords:** health expenditure, CO_2_ emissions, GDP growth, long run relation, bootstrap ARDL

## Abstract

Using annual time-series data over the period 1975–2017, the researcher applied the bootstrap autoregressive-distributed lag (ARDL) cointegration model developed by McNown et al. (1) to examine whether there is a long run relationship among health expenditure, CO_2_ emissions, and gross domestic product (GDP) per capita in 18 Organization for Economic Cooperation and Development (OECD) countries. We find cointegration exists in Netherlands when real GDP per capita serves as a dependent variable, in New Zealand when health expenditure is the dependent variable, and in the United States when CO_2_ emissions are dependent variables. The main results show evidence of a short run relationship between the three variables. The empirical results support that there is a bidirectional causality between health expenditure and GDP growth for Germany and the United States, between CO_2_ emissions and GDP growth for Canada, Germany, and the United States, and between health expenditure and CO_2_ emissions for New Zealand and Norway. The results also indicate that there are unidirectional causality in other countries.

## Introduction

Climate change caused by increased greenhouse gas emissions not only causes temperature rise but also affects global precipitation. Climate change has become an indisputable fact that poses a serious threat to the sustainable development of human survival, society, economy, and the environment (2). There is an important link between high concentrations of greenhouse gases and climate change that there is the impact of climate change on public health. In recent decades, however, the relationship among economic growth, environment deterioration, and health expenditure has received increasing attention in the literature. Economic growth, environmental degradation, and health care expenditure vary between these relationships—complex and important. Therefore, the negative externalities of low environmental quality due to the impact on human health expenditure are ignored (3).

Health is one of the most important factors to decide the quality of human capital. There are many factors that can affect the health status of the population, such as environmental health, socioeconomic status, economic development, and the environment quality. As environmental quality deteriorates, the deterioration of global environmental quality poses a serious challenge to healthy living. Particles from the burning of fossil fuels, sulfur dioxide, and CO_2_ emissions are a major contributor to global climate change, and this has been a topic of policy makers and a focus of researchers in many different fields of research (4).

Most of the literature in the past examined the pairwise relationship CO_2_ emissions, gross domestic product (GDP), and health expenditure. Over the past decade, the first empirical study is based on Grossman and Krueger (5), and a number of researchers have followed the Environmental Kuznets Curve (EKC) between economic growth and environmental quality (6–10). The second pairwise of empirical study shows that many researches have focused on the relationship between health expenditure and GDP (11–13). The majority of previous economists have studied to estimate the health care demand about the size of income elasticity and allocate health care resources. Finally, the third pairwise of empirical study was on the relationship between CO_2_ emissions and health expenditure (3, 14, 15). There are many researchers focusing on one-way causation from CO_2_ emissions to health expenditure and finding a positive relationship. However, only a limited number of empirical studies have concentrated on two-way causation between CO_2_ emissions and health expenditure.

CO_2_ emissions from Organization for Economic Cooperation and Development (OECD) countries account for half of global O_2_ emissions, yet CO_2_ emissions in OECD countries have been reduced since 2001. During this period, the OECD has made huge efforts to reduce CO_2_ emission intensity. CO_2_ emissions per unit of GDP decreased from 4.9 kg/USD in 2001 to 2.7 kg/USD in 2015. The share of CO_2_ emissions in global CO_2_ emissions also fell from 54% in 2001 to 38% in 2015 (13). Therefore, it is important to examine CO_2_ emissions for health expenditures and economic development in OECD countries.

This article aims to examine the health, CO_2_ emissions, and GDP growth nexus for 18 OECD countries by applying a bootstrap autoregressive-distributed lag (ARDL) test proposed by McNown et al. (1). This new method helps identify the occurrence of degenerate cases in ARDL tests. We, for the first time (compared to previous literature in OECD countries), used bootstrap models estimated by the ARDL test for long- and short-run relationships.

The main contribution of our research work to the existing studies is helpful for policy makers of OECD countries in particular and will provide some imperative understanding to other developed countries that include the 18 OECD countries. We used bootstrap ARDL approach for the long-run relationship and Granger causality tests to find directions of the links among the three variables. Therefore, this study aims at filling the gap in the literature by analyzing the health expenditure, CO_2_ emissions, and GDP growth using the newly proposed econometric methodology to test.

The study will be separated into five sections. Section Literature presents a review of the literature. The data and methods used are described in section Materials and Methods. Section Results presents the results. Section Discussion has discussions. Section Conclusion concludes the research.

## Literature

The purpose of this study was to demonstrate the interaction between CO_2_ emissions, health spending, and GDP growth in OECD countries. The pairwise correlation variables between the primary studies will be discussed.

### CO_2_ Emissions and GDP Growth

Most of the literature in the past concentrate on the relationship between environmental pollution and economic growth. First, the previous study was done to test the validity of the EKC hypothesis, which assumes that the relationship between economic growth and the environment is in the form of an inverted U. Selden and Song (9) used a nation's panel of data and the results confirm the inverted U. This hypothesis, however, was first proposed by Grossman and Krueger (5), where environmental degradation increases proportionally to income, then reaches a certain threshold when income reaches a stable point and then begins to decrease. Holtz-Eakin and Selden (16) examined marginal propensity to emit (MPE) and found that a diminishing MPE carbon dioxide as GDP per capita rises. Some authors (17–20) have reported the existence of the EKC hypothesis. Not all results support the ECK hypothesis. Cole et al. (21) have found evidence against the EKC hypothesis.

Using the cointegration technique of ARDL bound test and Graager causality, Halicioglu (22) examined the feedback hypothesis between CO_2_ emissions and economic expansion for Turkey. Saboori et al. (23) utilized ARDL bound approach to study the causal relationship between GDP growth and CO_2_ emissions for Malaysia and found unidirectional causality from GDP growth to CO_2_ emissions. Saboori and Sulaiman (24) determined a significant relationship between CO_2_ emissions and economic growth that supported the EKC hypothesis in Singapore and Thailand for the long run. Jebli et al. (25) found that two-way causalities existed in the long run between CO_2_ emissions and economic growth for 25 OECD countries. Cai et al. used the application of the newly introduced bootstrap ARDL bound test with structural breaks to examine the relationship among clean energy consumption, economic growth, and CO_2_ emissions. They found the existence of a long-term and short-term relationship between economic growth and CO_2_ emissions for the G7 nations (6).

### Health Expenditures and GDP Growth

The second part focuses on health expenditures and GDP growth. Much of the previous research has focused on estimating the impact of health care income elasticity on scale, except for distribution of health care resources.

Gerdtham and Löthgren test the cointegration between health expenditure and GDP in the 25 OECD nations for the period 1960–1997. The outcomes revealed that health expenditure and GDP are cointegrated for the 12 OECD nations (26). Baltagi and Moscone (27) also considered OECD countries using a panel of data to reconsider the long-run relationship between health care expenditure and income for the period 1971–2004. Their findings suggest that health care, a necessity, is in elastic demand. Wang applied the quantile panel-type analysis to determine the causality between health expenditure and economic growth from 1986 to 2007. The findings indicated that the estimation of quantile regression of the effect of health expenditure growth on economic growth is different (28). Albulescu et al. utilized health care spending for six EU member countries for the time period 1972–2013. The findings indicated that no significant convergence occurred with regard to the ratio of health care expenditure to GDP (29). Erçelik and Murthy and Okunade used ARDL to estimate the relationship between health expenditure and economic growth (30, 31).

### Health Expenditures and CO_2_ Emissions

The third research strand on the issue that has limited literature regarding the relationship between CO_2_ emissions and health expenditure discusses the relationship between environmental pollution (carbon emissions) and health expenditure.

Yazdi et al. (3) applied the ARDL approach to investigate carbon monoxide and sulfur oxide that have a positive relationship on health expenditures. Beatty and Shimshack used a cohort analysis to study relationships between carbon monoxide exposure and children's health outcome. They find that the increase in carbon monoxide resulted in positive and significant increases in children's health treatment (32). Boachie et al. in Ghana utilized health care expenditure for the time period 1970–2008. The finding revealed that a negative relationship exists between health care expenditure and CO_2_ emissions (14). According to the result presented in the study by Chaabouni et al. and Chaabouni and Saidi found that the low-income group countries have unidirectional causality from CO_2_ emissions to health expenditure (33, 34). Lu et al. (15) verified a negative effect of CO_2_ emissions and other pollutants on human health in 30 Chinese provinces.

Only a few studies have focused on the relationship among health expenditures, CO_2_ emissions, and economic growth. Chaabouni et al., Chaabouni and Saidi, and Chaabouni and Abdnnadher provide evidence of causality among health expenditures, CO_2_ emissions, and economic growth (33–35). Zaidi and Saidi showed a one-way causal relationship from health expenditure to GDP and a two-way causal relationship between health expenditure and CO_2_ emissions in sub-Saharan African countries (36). Ghorashi and Rad applied dynamic simultaneous equation model to investigate health expenditures, CO_2_ emissions, and economic growth in Iran. The empirical findings supported the presence of two-way causality flowing among CO_2_ emissions and economic growth and one-way causality from health expenditure to economic growth (37). Wang (28) used the ARDL model for the time period 1995–2017 to examine the dynamic linkages among CO_2_ emissions, health expenditures, and economic growth in Pakistan. The results of their empirical study show a long-run relationship among the three variables and a two-way relationship of causality between health expenditure and CO_2_ emissions and between health expenditure and economic growth, as well as a one-way causal relationship from CO_2_ emissions to health expenditure.

## Materials and Methods

This study covers 18 OECD countries between 1975 and 2017. The 18 OECD countries selected for this study are Australia, Austria, Belgium, Canada, Denmark, Finland, Germany, Ireland, Japan, Netherland, New Zealand, Norway, Portugal, Spain, Sweden, Turkey, the United Kingdom, and the United States. Real GDP per capita (GDP) are obtained from the World Development Indicators, World Bank. Per capita real health expenditure (HE) in U.S. PPP dollars older came from OECD Statistics. CO_2_ emissions (CO_2_) data are retrieved from The BP Statistical Review of World Energy (available at: http://www.bp.com/). All variables are transformed into natural logarithm form.

### Research Framework

The ARDL model is as follows:

(1) $$\begin{array}{r} y_{t}\;=\;c+\overset{k}{\underset{i=1}{\sum }}\alpha_{i}y_{t-i}+\overset{k}{\underset{j=0}{\sum }}\beta_{i}x_{t-i}+\overset{k}{\underset{j=0}{\sum }}\gamma_{i}z_{t-i}+\overset{p}{\underset{h=0}{\sum }}\lambda_{j}S_{t,j}+\mu_{t} \end{array}$$

*i* and *j* are the indicators of the lag period, *i* = 1, 2…, *k*; *j* = 0, 1…, *k; h* = 0, 1…, *p*. *t* represent time *t* = 1, 2,…, *T*. The *y*_*t*_ in the equation is the explanatory variable, *x*_*t*_ and *z*_*t*_ are the explanatory variables, and the variable *S*_*t, j*_ is a dummy variable. The parameters α_*i*_, β_*I*_, *and* γ_*i*_ are the coefficients of the lag of *y*_*i*_, *x*_*i*_, and γ_*i*_. The error term is μ_*t*_, and Equation (2) can be rewritten and expanded into the following equation:

(2) $$\begin{array}{l} \Delta y_{t}=c+\overset{k-1}{\underset{i=1}{\sum }}a_{1,i}\Delta y_{t-i}+\overset{k-1}{\underset{j=1}{\sum }}a_{2,i}\Delta x_{t-i}+\overset{k-1}{\underset{j=1}{\sum }}a_{3,i}\Delta z_{ti}\\ \;+\overset{p}{\underset{h=1}{\sum }}b_{j}D_{t,j}+b_{1}y_{t-1}+b_{2}x_{t-1}+b_{3}z_{t-1}+\varepsilon_{t} \end{array}$$

where $b_{1}=-1+\overset{k}{\underset{i=1}{\sum }}\alpha_{i},\;b_{2}=\overset{k}{\underset{i=0}{\sum }}\beta_{i},\;b_{3}=\overset{k}{\underset{i=0}{\sum }}\gamma_{i}$ and other parameters are the function values of the original parameters in Equation (1).

McNown et al. showed the bootstrap method to the ARDL tests of cointegration and proposed a cointegration that requires rejecting the *F*-test (denoted as *F*′) *H*_0_ : *b*_1_ = *b*_2_ = *b*_3_ = 0 and the other *F*-test (denoted as *F*″) *H*_0_ : *b*_2_ = *b*_3_ = 0; *t*-test on the lagged dependent variable *H*_0_ : *a*_1_ = 0 (28). They showed that two degenerate situations can arise. Situation 1 occurs when *F*′ and *t* are significant but *F*″ is not significant. In this situation, the joint significance of the error correction terms is due solely to the lag of the dependent variable. Thus, the explanatory variables are not part of the long-run cointegrating relation. The other situation, degenerate situation 2, occurs when *F*′and *F*″are significant but *t* is not significant. McNown et al. added a test on the lagged independent variables (*F*-test denoted as *F*″) to complement the existing *F*′and *t*-tests for cointegration as proposed by Pesaran et al. (1, 38).

McNown et al. showed that the bootstrap ARDL test has better size and power properties than the asymptotic test in the ARDL bound test. After testing the long-term relationships, we concluded that short-run causal relationship will be determined by standard Granger causality tests if there is no cointegration relationship among *y, x* and *z*. We used the Granger causality test for *x* and *z*, which should include the lagged difference on *x* or *z*, and we tested *a*_2, *j*_ = 0 or *a*_3, *j*_ = 0 in Equation (2). However, if there is cointegration between the dependent variable and the independent variable, they will form a fixed linear combination. In this case, the short-term relationship test should include the hysteresis difference of *x* or *z* and the hysteresis level of *x* or *z*, that is, test *a*_2, *j*_ and *b*_2_ or *a*_3, *j*_ and *b*_3_.

## Results

The ARDL bound test allows the variables with different orders of integration [I(0) or I(1), not for I(2)] (29). The Augmented Dickey–Fuller (ADF), Kwiatkowski-Phillips-Schmidt-Shin (KPSS), and Zivot-Andrews (ZA) unit root tests were employed to test the integration level (39–41). We found that all series have a unit root at 5% level of significanec, but all variables are stationary in the first differences when AD, KPSS, and ZA tests are used. To conserve space, the unit root test results are not shown here.

### Bootstrap ARDL Test (Long Run) With Structural Breaks

Table 1 provides the results of the Bootstrap ARDL tests. This study uses the two *F*-test ($F_{c}^{\;\prime }$ and $F_{c}^{\;\prime \prime }$) and one *T*-test (*t*_*c*_) as critical values for determining the long-run forcing variable found in McNown et al. We believe that only several estimated equations are able to reject the null hypothesis of the *t*-test and both *F*-tests simultaneously. We could only find cointegrated evidence for Netherlands when GDP serves as the dependent variable, for New Zealand when health expenditure is the dependent variable, and for the United States when CO_2_ emission is the dependent variable. Degenerated case 2 also occurs for Finland, where both the *F*′-test and *F*″-test are statistically significant but not the *t*-test. Besides, we believe that no cointegration can be revealed by bootstrap ARDL test among the GDP, health expenditures, and CO_2_ emissions for Australia, Austria, Belgium, Canada, Denmark, Germany, Ireland, Japan, Norway, Portugal, Spain, Sweden, Turkey, and the United Kingdom. Although not many countries show a long-run relationship between GDP, health expenditures, and CO_2_ emissions, however, some causality patterns are identified in the short run.

**Table 1:** Cointegration results: health expenditures (HE), CO2 emissions (CO_2_), and gross domestic product (GDP) growth.

**Country**	**DV|IV**	**Dummy variables**	***F*′**	**${\text{F}}_{c}^{\;\prime }$**	***t***	***t*_*C*_**	***F*^″^**	**${\text{F}}_{c}^{\;\prime \prime }$**	**Result**
Australia (1)	HE|GDP,CO _2_	D83, D93, D99, D12	2.223	3.826	−2.142	−2.787	3.330	3.439	Non-cointegration
	GDP|HE, CO_2_	D83, D05	−2.483	4.548	−2.606	−3.109	2.203	5.984	Non-cointegration
	CO_2_|GDP, HE	D81, D87, D94, D04	2.554	3.081	−1.246	−2.341	1.425	3.674	Non-cointegration
Austria (1)	HE|GDP,CO_2_	D87, D93, D04	0.628	2.509	−0.895	−1.249	0.588	2.787	Non-cointegration
	GDP|HE, CO_2_	D90, D04	3.388	3.767	−2.921	−2.434	3.535	4.072	Non-cointegration
	CO_2_|GDP, HE	D95	2.899	4.249	−1.981	−3.100	4.001	5.605	Non-cointegration
Belgium (2)	HE|GDP,CO_2_	D81, D90, D99, D08	1.498	2.874	−0.164	−1.751	1.899	3.164	Non-cointegration
	GDP|HE,CO_2_	D90, D04	3.060	3.473	−2.982	−2.453	3.302	4.168	Non-cointegration
	CO_2_|GDP, HE	D82, D90, D96, D11	0.664	3.569	−0.454	−2.404	0.341	4.368	Non-cointegration
Canada (2)	HE|GDP,CO_2_	D85, D91, D98, D06	1.267	3.151	−1.443	−2.300	1.373	3.654	Non-cointegration
	GDP|HE,CO_2_	D04	1.821	4.763	−2.301	−3.157	2.432	6.149	Non-cointegration
	CO_2_|GDP, HE	D88, D97	1.892	3.256	−1.340	−2.338	2.706	3.603	Non-cointegration
Denmark (1)	HE|GDP,CO_2_	D93, D01, D07	2.001	2.714	0.731	−1.839	2.226	2.919	Non-cointegration
	GDP|HE,CO_2_	D90, D04	3.744	4.467	−3.300	−3.042	4.850	5.121	Non-cointegration
	CO_2_|GDP, HE	D09	2.101	4.459	−1.982	−3.008	3.150	5.189	Non-cointegration
Finland (2)	HE|GDP,CO_2_	D81, D90, D03, D09	0.661	2.987	0.137	−2.174	0.985	3.488	Non-cointegration
	GDP|HE,CO_2_	D87, D03	3.032	3.288	**−2.372**	−2.238	**4.549**	3.471	Non-cointegration
	CO_2_|GDP, HE	D94, D12	2.086	4.336	0.284	−2.958	2.944	5.487	Non-cointegration
Germany (3)	HE|GDP,CO_2_	D84, D99, D08	2.052	2.786	−2.308	−1.402	2.434	2.973	Non-cointegration
	GDP|HE,CO_2_	D87, D04	2.198	2.906	−2.374	−1.739	2.016	2.921	Non-cointegration
	CO_2_|GDP, HE	D82, D92, D99, D07	2.528	3.044	−2.349	−2.019	3.480	3.232	Non-cointegration
Ireland (1)	HE|GDP,CO_2_	D95, D03, D09	2.644	4.269	−1.118	−2.719	3.517	5.372	Non-cointegration
	GDP|HE,CO_2_	D90, D96, D04	3.803	4.897	−3.108	−3.339	5.632	6.194	Non-cointegration
	CO_2_|GDP, HE	D86, D96, D02, D11	0.292	3.867	−0.129	−2.493	0.188	4.672	Non-cointegration
Japan (3)	HE|GDP,CO_2_	D83, D92, D00, D10	1.349	2.735	−0.674	−1.631	1.237	2.802	Non-cointegration
	GDP|HE,CO_2_	D81, D87, D93, D08	0.999	3.763	0.146	−2.259	0.451	3.625	Non-cointegration
	CO_2_|GDP, HE	D90, D96	2.243	4.986	−2.071	−3.329	3.339	6.571	Non-cointegration
Netherland (1)	HE|GDP,CO_2_	D81, D88, D99, D06	1.678	3.028	−0.338	−2.068	1.588	3.287	Non-cointegration
	GDP|HE,CO_2_	D90, D04	**7.207**	4.846	**−4.605**	−3.339	**9.427**	5.490	**Cointegration**
	CO_2_|GDP, HE	D91	0.632	3.593	−0.756	−2.478	0.617	4.229	Non-cointegration
New Zealand (2)	HE|GDP,CO_2_	D81, D97, D05	**5.012**	4.096	**−3.024**	−2.733	**7.260**	5.226	**Cointegration**
	GDP|HE,CO_2_	D82, D04	4.741	4.859	−3.258	−3.119	6.492	5.663	Non-cointegration
	CO_2_|GDP, HE	D84, D90, D01	1.829	3.117	1.015	−2.054	2.044	3.176	Non-cointegration
Norway (1)	HE|GDP,CO_2_	D86, D94, D02	0.566	2.631	−0.803	−1.410	0.284	2.799	Non-cointegration
	GDP|HE,CO_2_	D90, D03	2.195	3.717	−2.537	−2.694	2.261	3.718	Non-cointegration
	CO_2_|GDP, HE	D94	1.840	4.406	−2.046	−3.143	2.038	5.393	Non-cointegration
Portugal (1)	HE|GDP,CO_2_	D83, D00	2.703	3.824	−1.128	−2.766	2.043	3.992	Non-cointegration
	GDP|HE,CO_2_	D82, D04	2.612	5.095	−1.839	−3.453	0.200	5.401	Non-cointegration
	CO_2_|GDP, HE	D81, D89, D95, D10	1.844	3.040	−2.139	−2.305	0.855	3.406	Non-cointegration
Spain (1)	HE|GDP,CO_2_	D03	1.210	3.976	−0.123	−2.774	1.743	4.613	Non-cointegration
	GDP|HE,CO_2_	D81, D87, D04	2.651	4.876	−2.562	−3.307	0.915	5.901	Non-cointegration
	CO_2_|GDP, HE	D89, D97	0.249	4.127	−0.740	−2.880	0.018	4.998	Non-cointegration
Sweden (1)	HE|GDP,CO_2_	D86, D00, D06, D12	0.556	3.516	−0.896	−2.209	0.807	3.425	Non-cointegration
	GDP|HE,CO_2_	D82, D04	1.642	3.991	−2.216	−2.680	1.284	4.377	Non-cointegration
	CO_2_|GDP, HE	D81, D09	2.972	4.267	−1.989	−2.917	2.536	5.422	Non-cointegration
Turkey (1)	HE|GDP,CO_2_	D85, D94, D00	1.705	3.198	−1.821	−1.878	1.584	3.484	Non-cointegration
	GDP|HE,CO_2_	D84, D94, D00	2.177	3.922	−0.517	−1.187	0.718	4.817	Non-cointegration
	CO_2_|GDP, HE	D85, D95, D06, D12	0.622	2.759	0.039	−2.045	0.729	3.199	Non-cointegration
United Kingdom (2)	HE|GDP,CO_2_	D92, D02, D12	1.449	3.079	−1.085	−1.904	1.569	3.306	Non-cointegration
	GDP|HE,CO_2_	D88, D97, D03	2.512	4.279	−2.607	−2.978	3.286	5.391	Non-cointegration
	CO_2_|GDP, HE	D81, D09	0.801	4.059	−0.741	−2.619	1.065	5.365	Non-cointegration
United States (4)	HE|GDP,CO_2_	D83, D91, D02	2.134	5.569	−1.253	−3.145	1.148	7.243	Non-cointegration
	GDP|HE,CO_2_	D85, D96, D04	2.851	5.131	−2.617	−2.642	4.150	6.265	Non-cointegration
	CO_2_|GDP, HE	D88, D94, D00, D09	**4.599**	3.787	**−2.267**	−2.064	**6.838**	4.441	**Cointegration**

*The parentheses is optimal lag order based on Akaike Information Criterion (AIC). D84 means a dummy variable for the year 1984; other years are 0. F′ is the F-statistics for the coefficients of HE_t−1_, GDP_t−1_ and C_O_2_t−1_; t_dep is the t-statistics for the dependent variable, and F″ is the F-statistics for the independent variable. $F_{c}^{\;\prime }$, t_C_ and $F_{c}^{\;\prime \prime }$ are the critical values at the 10% significance level, generated from the bootstrap program. Bold value represents Cointegration results*.

### Short-Run Granger Causality Test

Based on the results from ARDL cointegration test, we test the causality among the variables for 18 OECD countries by standard Granger causality test to indicate the casual relationship among health expenditure, GDP growth, and CO_2_ emissions. From Table 2, we can see that a feedback exists between health expenditure and GDP, between health expenditure and CO_2_ emissions, and between CO_2_ emissions and GDP.

**Table 2:** Granger causality results: health expenditures, CO_2_ emissions, and gross domestic product (GDP) growth.

**Country**	**Variables**	**HE equation**	**GDP equation**	**CO_**2**_ equation**
		***F*-statics,** **(*p*-value) (Sign)**	***F*-statics,** **(*p*-value) (Sign)**	***F*-statics,** **(*p*-value) (Sign)**
Australia	Health expenditures	–	1.032 (0.310) (+)	−0.603 (0.537) (−)
	GDP	0.015 (0.988) (+)	–	−2.089 (0.000) ^**^(−)
	CO_2_	−0.780 (0.441) (−)	0.409 (0.685) (+)	–
Austria	health expenditures	–	0.209 (0.836) (+)	1.843 (0.074)^*^ (+)
	GDP	−1.325 (0.195) (−)	–	−0.434 (0.667) (−)
	CO_2_	0.540 (0.593) (+)	1.170 (0.251) (+)	–
Belgium	Health expenditures	–	0.059 (0.943) (−)	2.011 (0.154) (−)
	GDP	3.819 (0.035)^**^ (+)	–	4.235 (0.025)^**^ (−)
	CO_2_	0.696 (0.508) (+)	2.258 (0.123) (+)	–
Canada	Health expenditures	–	1.791 (0.185) (+)	2.372 (0.111) (+)
	GDP	0.791 (0.464) (−)	–	19.80 (0.000)^***^ (+)
	CO_2_	1.032 (0.370) (+)	3.325 (0.05)^**^ (+)	–
Denmark	Health expenditures	–	0.235 (0.816) (+)	1.227 (0.229) (+)
	GDP	2.079 (0.046)^**^ (+)	–	1.825 (0.077)^*^ (+)
	CO_2_	−1.427 (0.593) (−)	−1.501 (0.136) (−)	–
Finland	Health expenditures	–	1.108 (0.344) (−)	1.332 (0.280) (+)
	GDP	3.348 (0.050)^**^ (+)	–	1.117 (0.342) (+)
	CO_2_	1.039 (0.368) (+)	2.563 (0.095)^*^ (+)	–
Germany	Health expenditures	–	6.889 (0.001)^***^ (−)	1.771 (0.182) (+)
	GDP	4.454 (0.013)^**^ (−)	–	3.996 (0.020)^**^ (−)
	CO_2_	1.268 (0.295) (−)	4.894 (0.008)^***^ (+)	–
Ireland	Health expenditures	–	−0.151 (0.881) (−)	0.396 (0.695) (+)
	GDP	−0.789 (0.195) (−)	–	−0.374 (0.711) (−)
	CO_2_	2.120 (0.042)^**^ (+)	1.020 (0.315) (+)	–
Japan	Health expenditures	–	0.501 (0.685) (−)	1.579 (0.221) (−)
	GDP	0.492 (0.691) (+)	–	7.589 (0.001) ^***^(+)
	CO_2_	0.540 (0.793) (−)	0.498 (0.687) (−)	–
Netherland	Health expenditures	–	2.296 (0.117) (+)	−0.657 (0.516) (−)
	GDP	−0.061 (0.952) (−)	–	0.783 (0.439) (+)
	CO_2_	2.828 (0.008)^***^ (+)	4.464 (0.019) ^**^(+)	–
New Zealand	Health expenditures	–	1.321 (0.283) (+)	2.637 (0.089)^*^ (−)
	GDP	2.307 (0.099)^*^ (+)	–	0.394 (0.678) (+)
	CO_2_	5.622 (0.004)^***^ (+)	0.386 (0.683) (+)	–
Norway	Health expenditures	–	−0.467 (0.644) (−)	2.095 (0.044) ^**^(+)
	GDP	−1.648 (0.109) (−)	–	0.789 (0.436) (+)
	CO_2_	2.989 (0.005)^***^ (+)	0.347 (0.731) (+)	–
Portugal	Health expenditures	–	0.082 (0.935) (+)	1.237 (0.226) (+)
	GDP	5.028 (0.000) ^***^(+)	–	−0.679 (0.502) (−)
	CO_2_	−1.483 (0.148) (−)	1.007(0.321) (+)	–
Spain	Health expenditures	–	1.416 (0.259) (+)	1.087 (0.351) (+)
	GDP	3.657 (0.039)^**^ (+)	–	3.070 (0.062)^*^ (+)
	CO_2_	1.167 (0.326)(+)	2.240 (0.126) (+)	–
Sweden	Health expenditures	–	2.296 (0.117) (−)	2.507 (0.099) *(−)
	GDP	9.274 (0.000)^***^ (+)	–	0.363 (0.699) (−)
	CO_2_	1.013 (0.379) (−)	0.739 (0.487) (+)	–
Turkey	Health expenditures	–	1.291 (0.206) (+)	0.574 (0.570) (+)
	GDP	1.634 (0.112) (+)	–	0.133 (0.895) (+)
	CO_2_	−0.510 (0.6714) (−)	0.232 (0.818) (+)	–
United Kingdom	Health expenditures	–	1.604 (0.219) (−)	3.855 (0.033)^**^ (−)
	GDP	1.192 (0.319) (−)	–	3.116 (0.060)^*^ (−)
	CO_2_	0.502 (0.611) (+)	0.726 (0.493) (+)	–
United States	Health expenditures	**–**	4.591 (0.009) ^***^(+)	0.685 (0.612) (−)
	GDP	5.388 (0.005)^***^ (−)	–	2.604 (0.071) *(−)
	CO_2_	5.533 (0.004)^***^ (+)	3.644 (0.022) ^**^(−)	–

The asterisks ^***^, ^**^, and ^*^ denote the significance at the 1, 5, and 10% levels, respectively. Additionally, the parentheses are p-value and sign for the coefficients. The case of non-cointegration and its causality test involved only lagged differenced variables. Bold value represents Granger causality results.

We find a two-way Granger causality running between health expenditure and GDP for Germany and the United States. This finding supports the past empirical studies (42–44). The results from Granger causality tests indicate the existence of bidirectional causality between health expenditure and CO_2_ emissions for New Zealand and Norway. Studies look at the causal relationship between health expenditures and GDP growth that, in most of these countries, have bidirectional causality (35). We report the causal link between CO_2_ emissions and GDP growth for Canada, Germany, and the United States by employing a bootstrap ARDL approach. In the short running analysis, many studies also find evidence to support the two-way Granger causality between CO_2_ emissions and GDP growth (28, 37, 45, 46).

There was unidirectional causality running from GDP to health expenditures for Belgium, Denmark, Finland, New Zealand, Sweden, Portugal, and Spain. The short-run relationship Granger causality was found from health expenditure to CO_2_ emissions for Austria, Belgium, Sweden, and the United Kingdom, and from CO_2_ emissions to health expenditures in Germany, Ireland, Japan, Netherlands, and the United States, and these countries exhibit only unidirectional causality. In the short run, it is unidirectional causality. Granger causality tests indicate that CO_2_ emissions Granger causes GDP in Finland and Netherlands, and GDP Granger causes CO_2_ emissions in Australia, Denmark, Japan, Spain, Sweden, and the United Kingdom.

## Discussion

The overall results revealed that there is no long-run relationships in the 18 OECD countries. There is no long-run relationship among health expenditures, CO_2_ emissions, and GDP growth that we support Atay and Ergun that we get the same result in Turkey (40). Wang et al. found a long-run relationship among CO_2_ emissions, health expenditure, and GDP in Pakistan (47). The researchers examined health expenditure, environmental pollution (CO_2_ emissions; nitrous oxide emissions), and GDP growth and indicated the long-run relationship among them in the sub-Saharan African countries (36). We only report a long-run relationship among health expenditure, CO_2_ emissions, and GDP growth in Netherlands, New Zealand, and the United States. There is important available that examined the link between health expenditure, CO2 emissions, and GDP growth in Netherlands, New Zealand and the United States.

Furthermore, we also estimated the Granger causality based on the ARDL model. The short-run relationship among the three variables, the coefficient value for CO_2_ emissions, reports a positive and significant effect with health expenditures in Ireland, Netherlands, United States, New Zealand, and Norway that means CO_2_ emissions increase health expenditures. The important policy is that the government actively limits carbon emissions because environmental pollution will increase health expenditures. The coefficient for GDP growth is showing the positive and significant effect between GDP and health expenditures, which shows that in the short run, the increasing level of GDP increases health expenditures. GDP growth sacrifices health factors because economic development may cause environmental pollution, and low-quality human capital forms more health losses. However, there do exist negative relations with short-run relationship running from GDP growth to health expenditures for a number of countries, including Germany and the United States. This may be the reason of the negative link between GDP and health expenditure for the case of Germany and the United States because human capital and institutional quality are in good condition. When growth increases, people's investment in physical exercise reduces the expenditure for health.

There are negative relations with short-run relationship from health expenditures to GDP growth for Germany but positive relations for the United States. Increasing health expenditures is associated with low productivity of human being because health creates disturbance for the economic growth (48). Health expenditures cannot lead to social security, efficient resource allocation, and better economies of scale in Germany (37). The human capital stock is positive and statistically significantly affects GDP growth in the United States (34). CO_2_ emissions have a positive and significant relation to GDP growth in Finland, Netherlands, Canada, and Germany. These countries have a high intensity of CO_2_ emissions due to the use of fossil fuels and rapid GDP growth. However, energy consumption and CO_2_ emissions do not increase the GDP growth of the United States but have a negative effect.

This study has found that health expenditure has a significant and positive effect on CO_2_ emissions at the level of 5% in Austria and Norway. In addition, CO_2_ emissions are negatively and significantly affected by health expenditures in Belgium, Sweden, the United Kingdom, and New Zealand. There are two effects on health expenditures that affect carbon emissions. The effect of health expenditures is related to population growth, thus as long as population increases, the rise in energy consumption results in greater environment pollution (33). On the other hand, health expenditures increase people's awareness of pollution and reduce carbon emissions. The first effect is significantly larger than the second effect in Austria and Norway and vice versa in Belgium, the United Kingdom, and New Zealand. In addition, GDP has a positive and significant effect on CO2 emissions in Canada, Denmark, Japan, and Spain but a negative effect on CO_2_ emissions in the United Kingdom, Australia, Germany, and the United States.

## Conclusions

This study contributes to the literature in the dynamic relationship among CO_2_ emissions, health care expenditure, and GDP growth for the 18 OECD countries over the period of 1975–2017. We investigate the bidirectional causal relationship between health expenditure and GDP and between CO_2_ emissions and GDP and between health expenditure and CO_2_ emissions for the three ARDL models. The results show that the bootstrap ARDL analysis is useful in evaluating the effects of different socioeconomic scenarios in the context of different positions for health expenditure and environment policy in OECD countries.

Our study results showed that there is cointegration existing in Netherlands when real GDP per capita serves as the dependent variable, in New Zealand when health expenditure is the dependent variable, and in the United States when CO_2_ emission is the dependent variable. The short-run relationship exists in a unidirectional causality relationship from CO_2_ emissions to health expenditure for Germany, Ireland, Japan, Netherlands, and the United States and from health expenditure to CO_2_ emissions for Austria, Belgium, Sweden, and the United Kingdom. There is a bidirectional causality relationship between health expenditure and CO_2_ emissions in New Zealand and Norway. This empirical result supports previous researches (33, 34, 47). There is a unidirectional causality relationship from GDP to health expenditure for Belgium, Denmark, Finland, New Zealand, Sweden, Portugal, and Spain. There is a bidirectional causality relationship between GDP and health expenditure for Germany and the United States (42–44). It is unidirectional causality from CO_2_ emissions to GDP for Finland and Netherlands, and GDP Granger causes CO_2_ emissions for Australia, Belgium, Denmark, Japan, Spain, and the United Kingdom. Our study results showed the bidirectional causality relationship between Canada, Germany, and the United States (28, 37).

The most important policy of these OECD countries is to limit CO_2_ emissions. In addition, these countries should adopt measures and policies to protect the quality of the environment to reduce the occurrence of health diseases. Environmental quality as a contributing factor to increased health spending has been proven. Our results demonstrate a significant increase in health spending on CO_2_ emissions in Ireland, Netherlands, the United States, New Zealand, and Norway. In some countries, health factors are sacrificed because of GDP growth because economic development may cause environmental pollution and low-quality human capital will cause more health losses. For some economies, however, including Germany and the United States, Granger causality does have a negative correlation from GDP to health spending. This may be the reason for the negative relations between GDP and health expenditure in Germany and the United States, as institutional quality and human capital are in good condition. When growth increases, people's investment in physical exercise reduces health spending. Therefore, environmental degradation is becoming a contemporary issue, and we urgently need to reexamine health and environmental policies so that GDP growth should not increase at the expense of public health or the environment. Due to the consumption of fossil fuels by OECD countries, industrial and household natural resources contribute significantly to CO_2_ emissions. It seems that policy makers should study the need for investment to promote technology transfer to protect environment quality.

## Data Availability Statement

The datasets generated for this study are available on request to the corresponding author.

## Author Contributions

C-MW designed the research, performed the experiments, and wrote the paper. H-PH collected the data. FL analyzed the results. C-FW edited the paper.

### Conflict of Interest

The authors declare that the research was conducted in the absence of any commercial or financial relationships that could be construed as a potential conflict of interest.
